# XPS Study of Grafting Paramagnetic Ions onto the Surface of Detonation Nanodiamonds

**DOI:** 10.3390/nano15040260

**Published:** 2025-02-10

**Authors:** Alexander Panich, Natalya Froumin, Aleksandr Aleksenskii, Anastasiya Chizhikova

**Affiliations:** 1 Department of Physics, Ben-Gurion University of the Negev, P.O. Box 653, Beer-Sheva 8410501, Israel; 2 Ilse Katz Institute for Nanoscale Science and Technology, Ben-Gurion University of the Negev, P.O. Box 653, Beer-Sheva 84105, Israel; 3 Ioffe Institute, St. Petersburg 194021, Russiachizhikova@mail.ioffe.ru (A.C.)

**Keywords:** XPS, nanodiamonds, paramagnetic ions, grafting to surface, magnetic and non-magnetic state

## Abstract

Grafting of paramagnetic transition and rare earth metal ions onto the surface of detonation nanodiamonds (DNDs) was successfully implemented in the recent decade and opened new opportunities in the biomedical application of these compounds, particularly as novel contrast agents for magnetic resonance imaging. The grafting was studied mainly using EPR, NMR, and magnetic measurements. Such a highly surface-sensitive, quantitative, chemical analytic technique as X-ray photoelectron spectroscopy (XPS) was very rarely used. In this paper, we report the XPS study of grafting transition and rare-earth metal ions (Cu^2+^, Co^2+^, Mn^2+^, and Gd^3+^) onto the surface of DNDs. Binding energies for metal, carbon, oxygen, and nitrogen atoms were determined and attributed to the corresponding ion states and atomic groups. Comparing XPS and EPR findings, we showed that the developed synthesis route resulted in almost complete grafting of manganese and gadolinium atoms in the form of paramagnetic ions Mn^2+^ and Gd^3+^ to the diamond surface, while only 30% of the copper atoms on the surface are in the paramagnetic state Cu^2+^, and the rest 70% are in the non-magnetic Cu^+^ state. It was not possible to draw a similar conclusion regarding Co^2+^ ions due to the lack of data on the amount of these paramagnetic ions on the DND surface.

## 1. Introduction

Diamond nanoparticles produced by the detonation method (detonation nanodiamonds, DNDs) have attracted great interest due to their numerous applications and commercial availability [1,2,3,4,5,6,7,8,9,10,11,12,13,14,15,16,17,18,19]. Among the recent attractive areas of the nanodiamond applications, one could mention nanodiamonds as catalyst support substances including putting them to use as single-atom catalysts [20,21,22], and mostly as contrast agents in the magnetic resonance imaging (MRI) [23,24,25,26,27]. To implement the latter, paramagnetic ions were initially chemically bound to the surface of diamond nanoparticles, mainly in organogadolinium fragments conjugated to the ND surface [23,24,25,26,27]. In the last decade, the direct grafting of paramagnetic ions of transition and rare earth metals onto the DND surface has been successfully implemented [28,29,30,31,32,33,34,35,36,37]. Such grafting results in an increase in the relaxation rate of nuclear spins of carbon and hydrogen atoms in the DND structure [28,29,30,31,32,33,34]. Furthermore, it significantly accelerates both spin-lattice and spin-spin relaxation of hydrogen spins of the solvent in aqueous suspensions of metal-grafted DNDs [35,36,37]. The latter demonstrate very high relaxivity, an order of magnitude higher than that of Dotarem, used as a contrast agent in the clinics, and are therefore very promising as new contrast agents for magnetic resonance imaging (MRI) [35,36,37].

Ionic grafting is performed by mixing the aqueous suspensions of purified DNDs with aqueous solutions of copper, cobalt, manganese, and gadolinium salt nitrates containing paramagnetic ions of these elements [28,29,30,31,32,33]. It would seem that the more paramagnetic ions are grafted to the surface, the more effective the resulting contrast agent should be. However, it has been established that the grafting of such ions is limited. The matter is that transition and rare earth ions are coupled to the DND surface via exchange with protons of closely located surface carboxyl (−COOH) groups [28,29,30,31,32,33,34], and the incorporation of ions is limited by the number of corresponding surface COOH groups. It should be noted that among the hydrocarbon and hydroxyl groups that each nanodiamond surface possesses, the number of carboxyl groups is not very large. Estimates by Comet et al. [38] showed that a DND particle with a diameter of 5 nm exhibits ∼67 −COOH groups. This means that no more than 33 doubly charged ions (or less than 22 triply charged ones) can be grafted to such a particle. Magnetic (SQUID) measurement data [31,39,40] indicate the presence of ∼18 Gd^3+^ ions incorporated into the surface of a 5 nm DND particle at a maximum nominal Gd concentration of ∼3.28 wt%. This means that not all metal atoms would be grafted to the DND surface, and increasing the metal concentration in the solution would not necessarily result in an increase in the grafted ions. Moreover, the question is whether all these metals are in paramagnetic Cu^2+^, Co^2+^, Mn^2+^ and Gd^3+^ states. For example, it has been recently suggested [41,42] that grafting DNDs with copper may result not only in DNDs with surface paramagnetic Cu^2+^ ions but also in DND complexes with diamagnetic Cu^+^ ions and even in neutral clusters of several Cu^0^ atoms located in the space between DND particles. Their fixation on the DND surface (if it exists) is unknown. XPS shows from 39 to 64 Cu atoms on the surface of a 5 nm DND particle [41], while EPR shows only 20 Cu^2+^ ions in such a particle [42]. The difference may be due to the presence of the above-mentioned diamagnetic ions and copper atoms.

To address this issue, in this paper, we investigated DNDs grafted with paramagnetic Cu^2+^, Co^2+^, Mn^2+^, and Gd^3+^ ions using X-ray photoelectron spectroscopy (XPS). XPS is a highly surface-sensitive quantitative chemical analysis technique that can be used to address a wide range of material problems. The sampling depth for XPS measurements is 5–10 nm from the top of the surface, which can in principle cover the entire volume of a DND particle with a diameter of 4.5–5 nm. We analyze the formation of metal complexes on the DND surface and consider them with the published EPR findings [28,29,30,31,32] and recent theoretical calculations on the fixation of transition metal ions on the surface of DND particles.

## 2. Materials and Methods

### 2.1. Samples

The initial nanodiamond material (DND powder) was repeatedly purified in boiled hydrochloric acid, then washed in boiled water [28,29,30,31,32,33,34] for removal of non-diamond *sp*^2^ carbon, iron-containing complexes and other para- and ferromagnetic impurities. This was carefully controlled for impurities using NMR, EPR and superconducting quantum interference device (SQUID) [30,31,32,43], and then precisely chemically modified with transition metal ions at Ioffe Institute (St. Petersburg, Russia). Chemical modification of the nanodiamond surface was done by mixing a suspension of DND particles with a water solution of copper acetate, cobalt or gadolinium nitrate, and manganese sulfate, respectively [28,29,30,31,32,33,34,35,36,37,44,45]. The concentration of DND suspension was about 0.5 wt%, and the concentration of a metal salt solution was 0.1 M.

The resulting mixture was subjected to intense and prolonged ultrasonic irradiation, which promoted disaggregation of DND particles and ion exchange in an aqueous solution between two- or three-charged metal ions (Cu^2+^, Co^2+^, Mn^2+^ or Gd^3+^) and two or three protons of the nearest carboxyl groups, terminated on the DND surface in many places. As a result, transition or rare earth metal ions form a metal-DND complex with the nanodiamond surface. This has repeatedly been experimentally confirmed [29,30,31,32,33,34,35,36,37,39,40,41,42,44,45]. After synthesis, the powder was removed from the suspension and dried. The number of carboxyl groups on the nanodiamond surface is about 60 [38] and in the case of trivalent ions, up to 20 metal ions could be bound with the nanodiamond particle surface. This number has been confirmed in experiments using electron paramagnetic resonance and static magnetization measurement [39]. The examined highly purified DNDs grafted with copper, cobalt, manganese, and gadolinium were studied, as summarized in Table 1. The metal content (wt.%) in the DND particle was determined by energy-dispersive X-ray analysis [28,29,30,31,32,33,34].

The average particle size in the suspension, determined by dynamic light scattering (DLS), ranged from 4.5 to 5 nm [28,29,30,31,32,33,34]. The interaction of hydrogen atoms of the surface carboxyl groups of a DND particle with metal ions leads to a decrease in the surface charge of the nanodiamond particle, resulting in a reduction of the z-potential of the surface (to −20 mV ÷ −15 mV in the case of Co^2+^, Cu^2+^, Mn^2+^ and almost to zero in the case of Gd^3+^ [45].

### 2.2. XPS Measurements

XPS data were collected using an ESCALAB -Xi+ ultra-high vacuum (5 × 10^−10^ mbar) X-ray photoelectron spectrometer equipped with an AlKα X-ray source and a monochromator. The X-ray beam size was 500 μm. Survey spectra were recorded with a pass energy of 150 eV, and high-energy resolution spectra were recorded with a pass energy of 20 eV. To correct for charging effects, all spectra were calibrated relative to the carbon C 1s peak positioned at 284.8 eV. Atomic ratios were calculated from peak intensity ratios and reported atomic sensitivity factors.

The AVANTAGE v6.6.0 Build 00114 Thermo Fisher Scientific software was used for data acquisition and analysis. For identification of the element’s chemical state, high-energy resolution measurements of the Cu2p Co2p, Mn2p, Gd3d, and Gd4d lines were performed with a pass energy of 50eV and a step size of 0.1 eV. For band deconvolution and fitting, the corresponding peaks for each element were fitted by using Lorentzian/Gaussian 30% function mixture and taking FWHM (full width at half maximum) in the range 1–2.5 eV. The XPS spectra were recorded at different acquisition times to get XPS high-resolution spectra. For Cu, Mn, and Co, electrons from the 2p level were measured, and for Gd, electrons from the 4d and 3d levels were measured. All these levels show spin-orbit splitting, which results in two states with different binding energies. Relative intensities were calculated taking into account sensitivity factors for each element.

## 3. Results and Discussion

Let us first discuss our XPS data on paramagnetic ions listed in Table 2, together with the data received from EPR and SQUID measurements.

### 3.1. XPS of Cu-DND Powder

**Figure 1:**
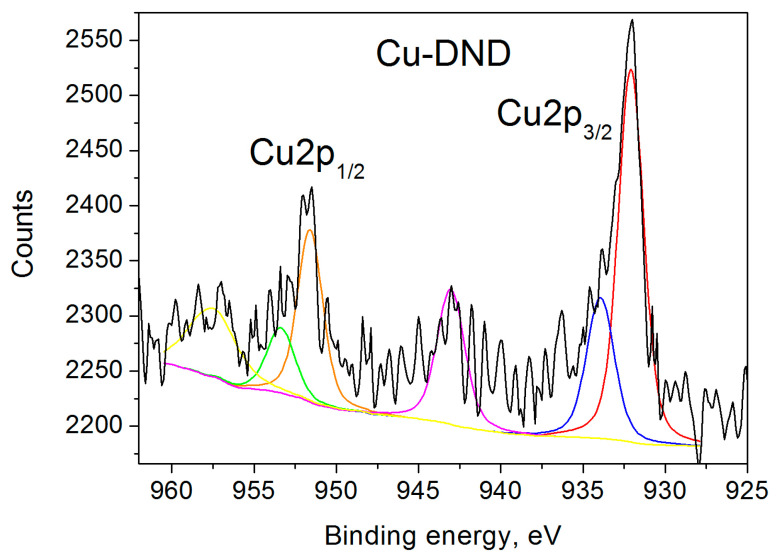
Cu 2p XPS spectra of Cu-DND sample. The black line is the experimental spectrum. Deconvolutions into two components are shown by red and blue lines for 2*p*_3/2_ bands and orange and green for 2*p*_1/2_ bands, correspondingly. The satellites are shown by magenta and yellow lines.

The recent literature XPS data for copper-grafted DNDs [41,42] show two bands determined by the spin-orbit splitting of the Cu 2p level (Δ ~ 19.8 eV). The symmetric 2p_1/2_ peak was observed at the binding energy of 952.7 eV, while the asymmetric 2p_3/2_ peak appeared to be composed of two products at the binding energies of 932.6 and 935 eV, attributed to Cu^+^ and Cu^2+^ ions, respectively. Our Cu 2p_3/2_ XPS spectrum (Figure 1) shows two bands at the binding energies of 932.09 eV and 933.94 eV, attributed to the non-magnetic Cu^+^ and paramagnetic Cu^2+^ ions, respectively [46,47,48,49,50,51,52]. 2p_1/2_ XPS spectrum (Figure 1) also shows two bands at the binding energies of 951.6 eV and 953.37 eV, assigned to the aforementioned Cu^+^ and Cu^2+^ ions [46,47,48,49,50,51,52]. The spectra also show two satellites of the above lines at 943.04 and 957.51 eV, correspondingly.

One can find from Table 2 that the atomic percentage of the paramagnetic divalent copper ions measured by XPS and the atomic percent of the paramagnetic Cu^2+^ ions measured by EPR practically coincide within the experimental errors. Thus we can conclude that around 30% of the grafted copper ions are paramagnetic and 70% are diamagnetic. The previous studies of Cu-DNDs mentioned above [41,42] do not provide percentages of Cu^+^ and Cu^2+^ ions, but their XPS spectra are very similar to ours.

**Figure 2:**
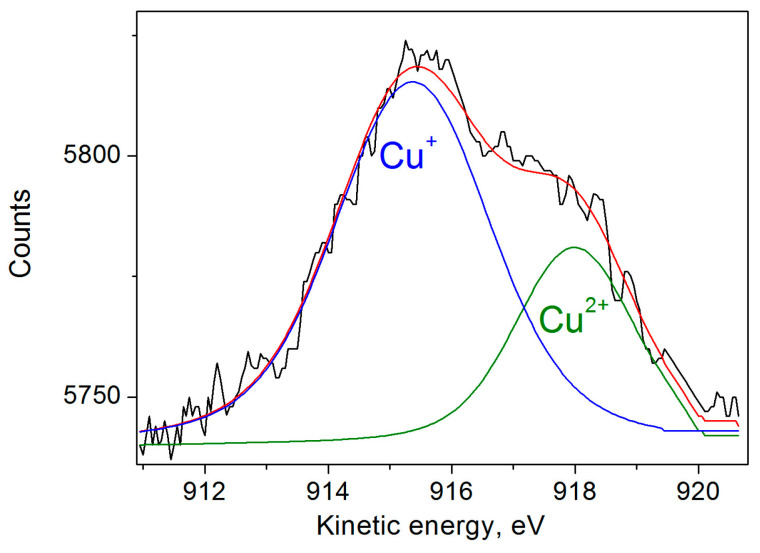
Auger spectrum of Cu-DND particles. Deconvolutions into two components is shown by blue and olive lines.

In addition, we also measured the Auger spectra of copper (Figure 2), which show two peaks of kinetic energy, Cu LM2 at 917.99 eV and Cu LM2 at 915.37 with atomic % of 29.41 and 70.59%, corresponding to Cu^2+^ and Cu^+^ bands. The data are in good agreement with the XPS and EPR results.

Grafting of paramagnetic ions to nanodiamonds utilizes the exchange between protons of surface carboxyl groups and charged copper ions [28,29,30,31,32,33]. The presence of Cu^+^ on the diamond surface means a 70% reduction of Cu^2+^ in the parent copper acetate to Cu^+^ in Cu-DND occurs.

### 3.2. XPS of Co-DND Powder

**Figure 3:**
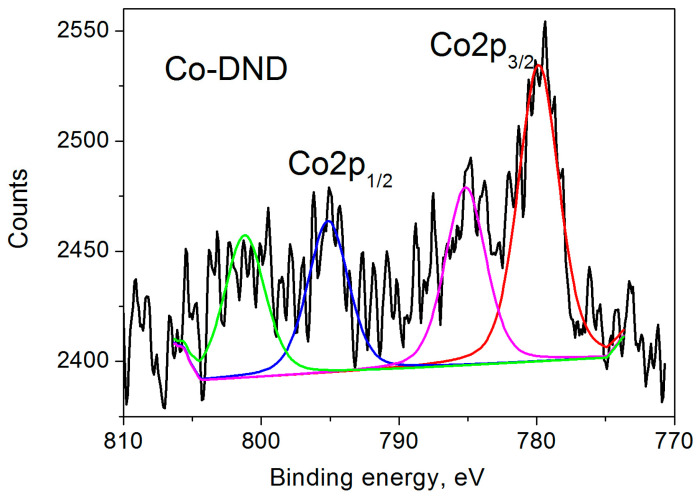
Co 2p XPS spectrum of Co-DND sample. 2*p*_3/2_ and 2*p*_1/2_ bands are shown by red and blue lines, and satellites are shown by magenta and green lines.

The XPS spectrum of Co-DND is shown in Figure 3. It consists of two main spin-orbit split bands (Co2p_3/2_ at 779.86 eV and Co2p_1/2_ at 795.15 eV, Δ = 15.3 eV) and two satellites at 785.16 and 801.20 eV for the above bands, respectively [50,53]. The binding energies of 779.86 eV and 795.15 eV are characteristic of the Co2p_3/2_ and Co2p_1/2_ bands of divalent Co^2+^ ions in CoO [47]. Close to these binding energy values are also observed in the oxides Co_2_O_3_ and Co_3_O_4_. Herewith, it is generally accepted that the energy gap between the main Co 2p peak and the satellite peak is closely related to the oxidation states. When the energy gap is ∼6.0 eV, the valence of the Co cation is assigned to 2+. If this energy gap is 9–10 eV, the spectrum is associated with Co cations having a valence of 3+ [54,55]. Our XPS data show the above gap as 5.3 and 6.05 eV for Co2p_3/2_ and Co2p_1/2_, respectively, confirming the existence of Co^2+^ ions in our sample, as previously obtained by EPR measurements [28].

Unfortunately, it was not possible to determine the Co^2+^ ion content in our Co-DND sample using EPR measurements [28]. Therefore, we cannot give a quantitative correspondence between the cobalt ions measured by XPS and EPR methods. However, both methods clearly show the presence of Co^2+^ ions on the surface of our Co-DND sample.

### 3.3. XPS of Mn-DND Powder

**Figure 4:**
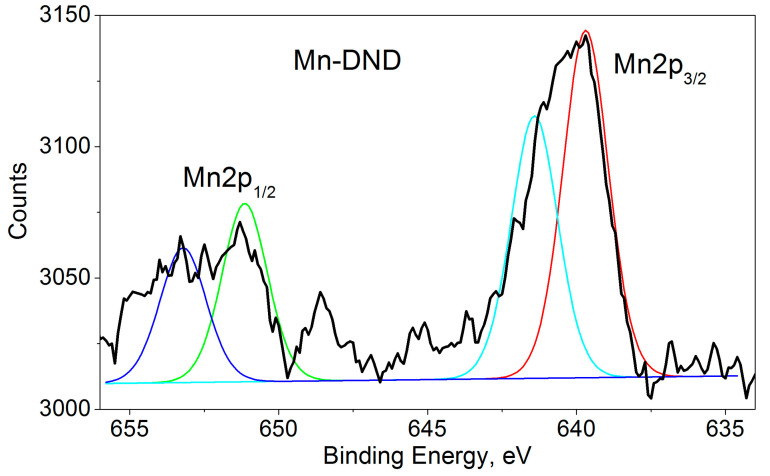
Mn 2p XPS of Mn-DND sample. Deconvolutions of the 2*p*_1/2_ and 2*p*_3/2_ bands into two components are shown by red, cyan, green, and blue lines.

The XPS spectra of our Mn-DND sample (Figure 4) show binding energies of 639.7 and 641.4 eV for Mn2p_3/2_ and 651.1 and 653.2 eV for Mn2p_1/2_, both characteristic of Mn2p transitions with a separation of 11.5–11.8 eV between the spin-orbit split primary peaks. Each band shows a two-component structure. The data included in the XPS database [47] do not show satellite peaks for Mn, so it can be assumed that the doublet detection is caused by nonequivalent manganese ions. The non-equivalence can be caused by different valences of the ions or different atomic configurations of the neighboring oxygen ions.

Note that manganese has six oxidation states (0, II, III, IV, VI, and VIII) with overlapping binding energy ranges, which poses a serious challenge for qualitative and quantitative analysis. Numerous XPS studies of Mn oxidation demonstrate difficulties in differentiating between the contributions of Mn^2+^, Mn^3+^, and Mn^4+^ ions [56,57,58,59,60], since similar binding energies are obtained for the oxides MnO, Mn_2_O_3_, Mn_3_O_4_, and MnO_2_, and the separation of the peaks leaves much to be desired. This is also true for our case.

We address this issue by comparing the manganese content of our sample, as determined from the XPS measurements presented here (0.07 at%), and previous EPR data showing 0.085 at% of Mn^2+^ ions [32] (Table 2). These numbers are virtually identical within experimental errors, leading us to conclude that all the manganese is Mn^2+^, located in various atomic configurations of neighboring oxygen ions.

### 3.4. XPS of Gd-DND Powder

**Figure 5:**
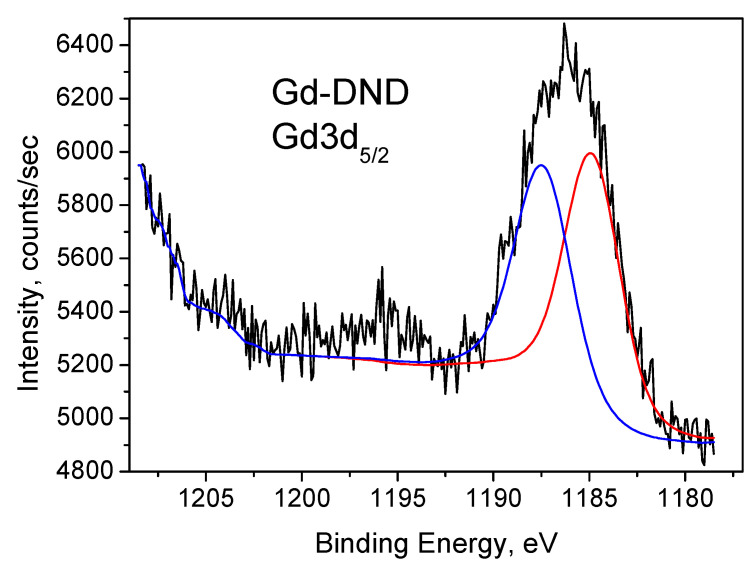
Gd3d_5/2_ XPS of Gd-DND sample. Deconvolution into two components is shown by red and blue lines.

**Figure 6:**
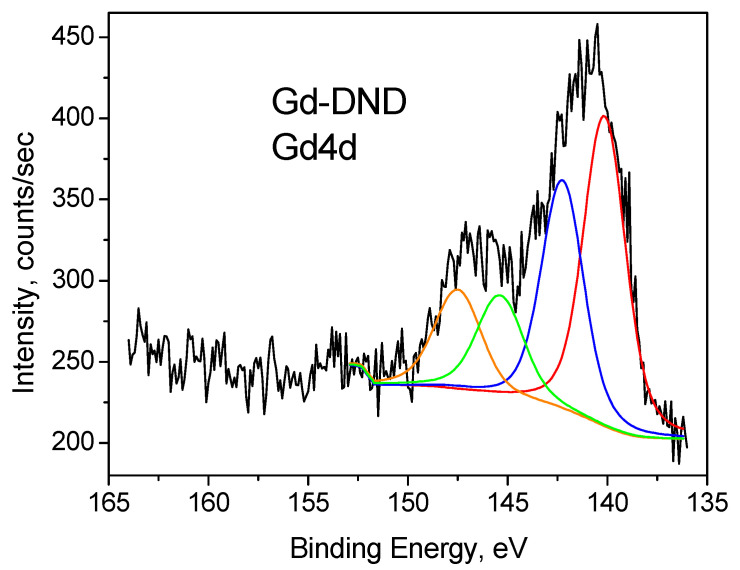
Gd4d XPS spectrum of Gd-DND sample. Deconvolution into four components is shown by red, blue, green and orange lines.

The results of our XPS measurements of Gd-DND powder are shown in Figure 5 and Figure 6. According to the literature [61,62,63,64,65,66,67,68,69], the Gd 3d core level spectra are split by spin-orbital coupling at the doublet (1187.3 eV for the 3d_5/2_ part and 1220 eV for the 3d_3/2_ part) with an energy difference of Δ = 32.7 eV. Note that we could not measure the Gd3d_3/2_ band with our equipment because it overlapped with the carbon Auger band and was hidden by it. The 3d_5/2_ band we measured is shown in Figure 5. One can find that this band consists of two components observed at 1185.4 and 1187.9 eV, which originate from two inequivalent Gd atoms.

Similarly, the Gd 4d core level spectra are split by the spin-orbit coupling into Gd4d_5/2_ and Gd4d_3/2_ bands with an energy difference of Δ = 5.3–5.4 eV (Figure 6). Each of these bands consists of two components, namely 139.9 eV and 142.2 eV for the 4d_5/2_ band and 145.2 eV and 147.5 eV for the 4d_3/2_ band. This finding is in good qualitative agreement with what is reported in the literature [61,62,63,64,65,66,67,68,69] and with our measurements of Gd_2_O_3_ powder, which contains only Gd^3+^ ions.

The way the sample was prepared should result in the formation of Gd^3+^ ions. This option is also supported by the fact that the atomic percentage of gadolinium measured by XPS is almost identical to that of paramagnetic Gd^3+^ ions measured by EPR (Table 2). Therefore the obtained bands refer to two nonequivalent Gd^3+^ ions bound to oxygen atoms and having different specific configurations.

To this end, recent DFT calculations [70,71] revealed two possible configurations of the Gd^3+^ chelate complexes on the diamond (111) surface. For the first configuration, the distances between the gadolinium atom and the three nearest oxygen atoms are 0.2281, 0.2182, and 0.2126 nm, respectively. For the second configuration of the Gd^3+^ chelate complex, the calculated distances between Gd and the three nearest oxygen atoms are 0.2281, 0.2384, and 0.2515 nm, and the distances to the other three more distant oxygen atoms are 0.2553, 0.2665, and 0.3588 nm, respectively.

### 3.5. C1s, N1s and O1s XPS

All XPS spectra of carbon, nitrogen and oxygen atoms and their quantization are given in Supplementary Materials.

## 4. Conclusions

In this paper, we report on the study of grafting transition and rare earth metal ions (Cu^2+^, Co^2+^, Mn^2+^, and Gd^3+^) to the surface of detonation nanodiamonds using the X-ray photoelectron spectroscopy (XPS) technique. The binding energies of metal, carbon, oxygen, and nitrogen atoms were determined and assigned to the corresponding ionic states and atomic groups. By analyzing the XPS data and comparing them with the EPR findings, we showed that the developed synthesis route leads to almost complete grafting of manganese and gadolinium atoms in the form of paramagnetic ions Mn^2+^ and Gd^3+^ to the diamond surface, while only 30% of the copper atoms on the surface are in the paramagnetic state Cu^2+^, and the rest 70% are in the non-magnetic Cu^+^ state.

## Figures and Tables

**Table 1:** List of samples examined. *S* is the electron spin of the paramagnetic ion, *N_A_* is the weighting percent of the transition and rare-earth elements in the DND particle, *N* is the number of paramagnetic ions per DND particle, and *N_S_* is the paramagnetic ion concentration.

Compound	Ion	*S*	*N_A_*, wt%	*N*	*N_S_*, spin/g	Ref.
Cu-DND	Cu^2+^ (3d^9^)	1/2	0.6	4	1.67 × 10^19^	[28]
Co-DND	Co^2+^ (3d^7^)	1/2	0.6	-	-	[28]
Mn-DND	Mn^2+^ (3d^5^)	5/2	0.12	8–9	4.1 × 10^19^	[32]
Gd-DND	Gd^3+^ (4f^7^)	7/2	3.28	18	7.85 ×10^19^	[31]

**Table 2:** XPS bands of paramagnetic ions in Cu-, Co-, Mn- and Gd-DNDs. Here BE is the binding energy, and at% is the atomic percent according to the XPS, EPR and SQUID measurements.

Compound	at%—XPS *	BE, eV	at%, XPS Components	at%—EPR *, SQUID
Cu-DND Cu2p3	0.14	932.63	100	
Cu^+^	0.098	932.09	70	
Cu^2+^	0.042	933.94	30	0.034
Co-DND Co2p3	0.13	780.9	52.9	-
Co-DND Co2p1		796.4	52.9	-
		801.9	47.1	
Mn-DND Mn2p3	0.07	639.7	56.3	0.085
		641.4	43.7	
Mn-DND Mn2p1		651.1	56.3	
		653.2	43.7	
Gd-DND Gd3d5	0.19	1185.4	54.4	0.164
		1187.9	45.6	
Gd-DND Gd4d	0.14	139.9	70. 5	0.164
		142.2	29.55	
		145.2	70.45	
		147.5	29.6	

* Due to the small number of paramagnetic ions, the typical experimental error in determining their atomic percentage is about 20% for XPS measurements and 15% for EPR measurements.

## Data Availability

Data are contained within the article.

## Supplementary Materials

The following supporting information can be downloaded at: https://www.mdpi.com/article/10.3390/nano15040260/s1.
