# Supplementary material for: XPS Study of Grafting Paramagnetic Ions onto the Surface of Detonation Nanodiamonds
Source: Nanomaterials (Basel). 2025 Feb 10;15(4):260. doi: 10.3390/nano15040260 (PMC11858398; doi:10.3390/nano15040260)
Supplement: Supplementary file 1 [file nanomaterials-15-00260-s001.zip › nanomaterials-3418639-supplementary.pdf]

Supplementary information

XPS study of grafting paramagnetic ions onto the surface of detonation nanodiamonds

Alexander M. Panich[1*], Natalya Froumin[2], Aleksandr E. Aleksenskii[3], Anastasia S. Chizhikova[3]

[1] *Department of Physics, Ben-Gurion University of the Negev, P.O. Box 653, Beer-Sheva 8410501, Israel*
[2] *Ilse Katz Institute for Nanoscale Science and Technology, Ben-Gurion University of the Negev, P.O. Box 653, Beer-Sheva 84105, Israel*
[3] *Ioffe Institute, Saint Petersburg, Russia*

Assignment of C1s, N1s, and O1s bands and relative abundances of the components in the initial and ion-grafted DND samples.

All XPS spectra of paramagnetic ions and the corresponding discussion are included in the main manuscript. Here we provide information on the carbon, nitrogen, and oxygen XPS spectra and the quantification of the corresponding functional groups in the initial and Cu-, Co-, Mn-, and Gd-grafted DNDs. The assignment of functional groups according to their binding energies follows those accepted in the literature [1-12].

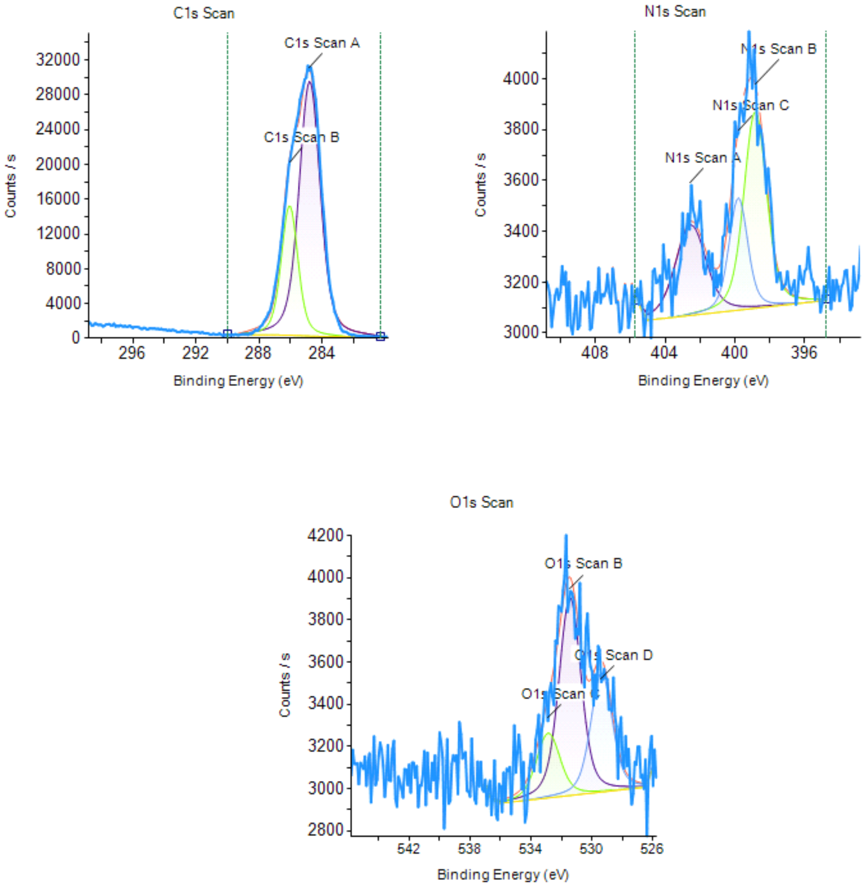

Figure S1. C1s, N1s, and O1s XPS spectra of initial DND sample for Cu-DND.

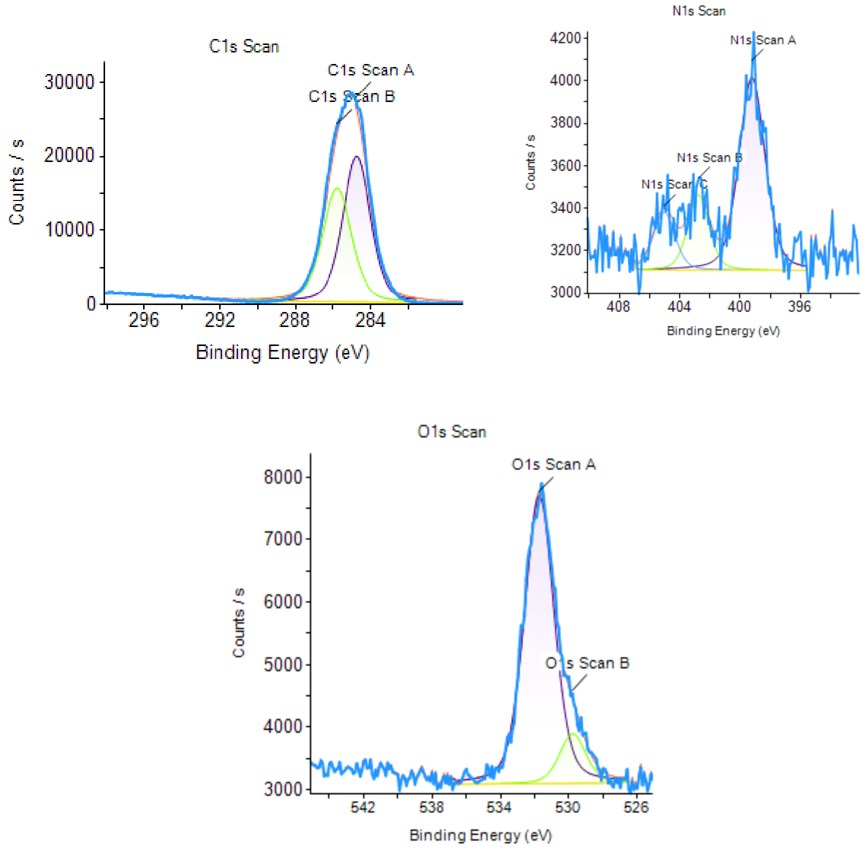

Figure S2. C1s, N1s, and O1s XPS spectra of Cu-DND sample.

Table S1. Elemental ID and Quantification in initial and Cu-DND samples

Sample	***Level***	***Peak BE***	***Atomic %***
**Initial DND**	C1s	284.8	96.2
	N1s	399.1	2.1
	O1s	531.6	1.7
**Cu-DND**	C1s	284.8	91.2
	Cu2p	931.9	0.1
	N1s	398.9	2.7
	O1s	531.4	6.0

Table S2. Detail Quantification of C1s, O1s, and N1s peaks in initial and Cu-DND samples

Compound	***Peaks***	***At%***	***Atomic group***	***Reference***
	C1s peaks	***C1s, at%***	***Group***	
Initial DND	284.8	71.2	C-C sp^3	[1-6]
	286.1	28.8	C-O	
Cu-DND	284.8	54.8	C-C sp^3	
	285.8	45.2	C-C, C-N, C-H	
	O1s peaks	***O1s, at%***	***Group***	[1-6]
Initial DND	529.4	30.05	COOH	
	531.5	51.71	C=O carbonyl	
	532.9	18.24	C-O-C, C-OH	
Cu-DND	529.9	13.28	COOH	
	531.7	86.72	C=O carbonyl	
	N1s peaks	***N1s, at%***	***Group***	[7-11]
Initial DND	398.8	47.8	N-sp^3C	
	399.8	22.6	N-H, C=N-C	
	402.5	29.6	N-O	
Cu-DND	399.2	63.4	N-sp^3C	
	402.8	20.5	N-O	
	405.0	16.1	shake-up peak, π-excitation	

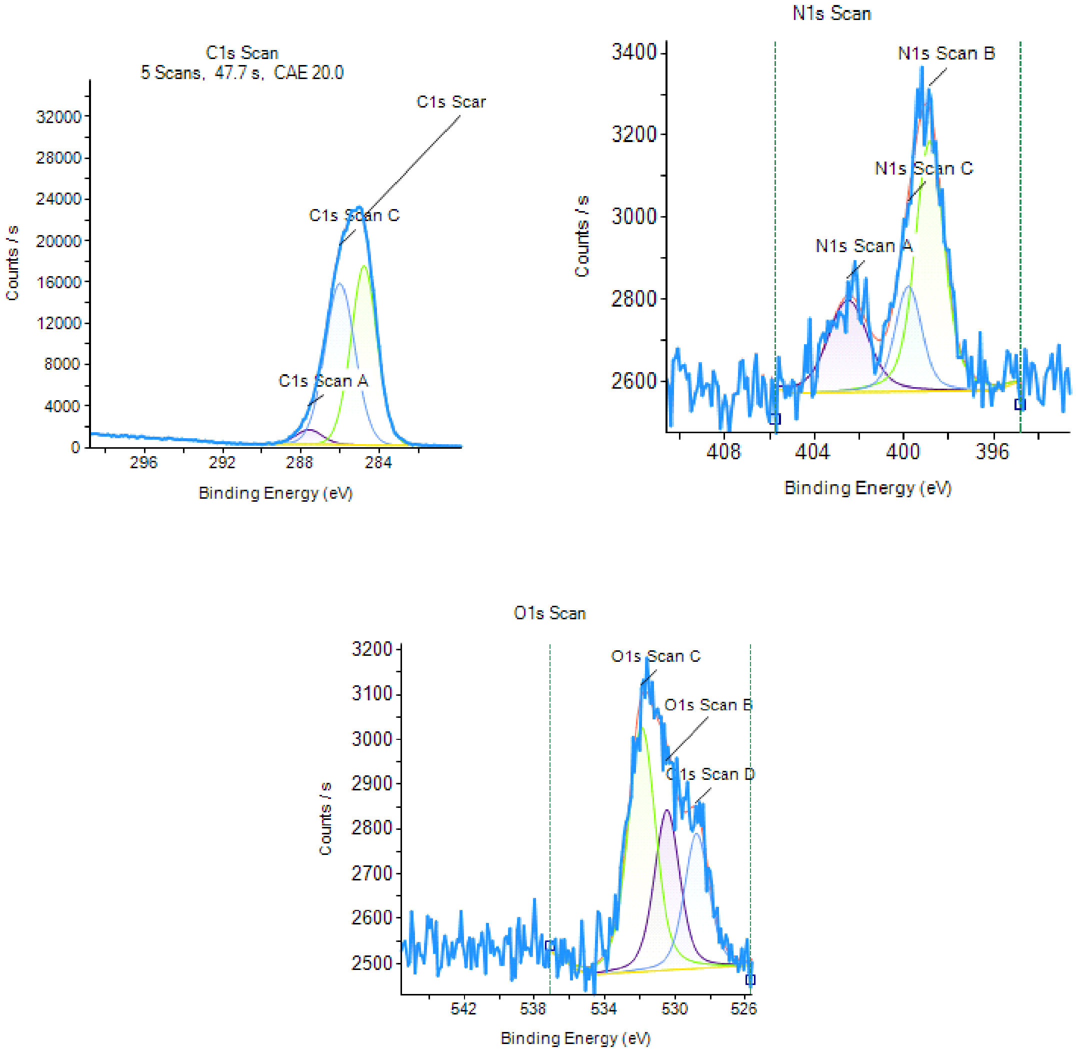

Figure S3. C1s, N1s, and O1s XPS spectra of initial DND sample for Co-DND.

Table S3. Elemental ID and Quantification in initial and Co-DND samples.

Sample	***Level***	***Peak BE***	***Atomic %***
**Initial DND**	C1s	284.8	96.6
	N1s	398.9	2.0
	O1s	531.3	1.4
**Co-DND**	C1s	284.8	91.5
	Co2p	779.4	0.1
	N1s	398.8	2.3
	O1s	531.6	6.1

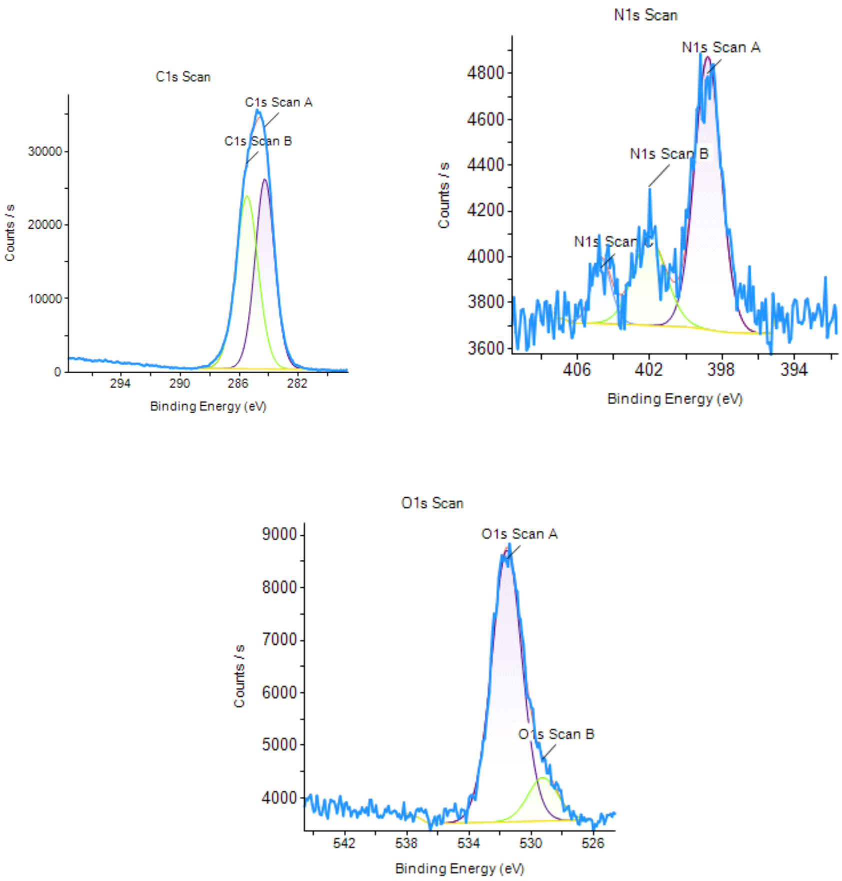

Figure S4. C1s, N1s, and O1s XPS spectra of Co-DND sample.

Table S4. Detail Quantification of C1s, O1s, and N1s peaks in initial DND and Co-DND

Compound	***C1s peak***	***C1s, at%***	***Group***	***References***
Initial DND	284.8	47.4	C-C sp^3	[1-6]
	286.0	48.6	C-O	
	287.6	3.9	C=O, N-C=O	
Co-DND	284.8	49.0	C-C sp^3	
	285.9	51.0	C-C, C-N, C-H	
	O1s peak	***O1s, at%***	***Group***	[1-6]
Initial DND	529.0	29.3	COOH	
	531.5	70.7	C=O carbonyl	
Co-DND	529.3	13.6	COOH	
	531.6	86.4	C=O carbonyl	
	N1s peak	***N1s, at%***	***Group***	[7-11]
Initial DND	398.9	52.8	N-sp^3C	
	399.8	20.4	N-H, C=N-C	
	402.5	26.8	N-O	
Co-DND	398.8	63.6	N-sp^3C	
	402.0	25.3	N-O	
	404.7	11.1	Shake-up peak, π-π^* excitation	

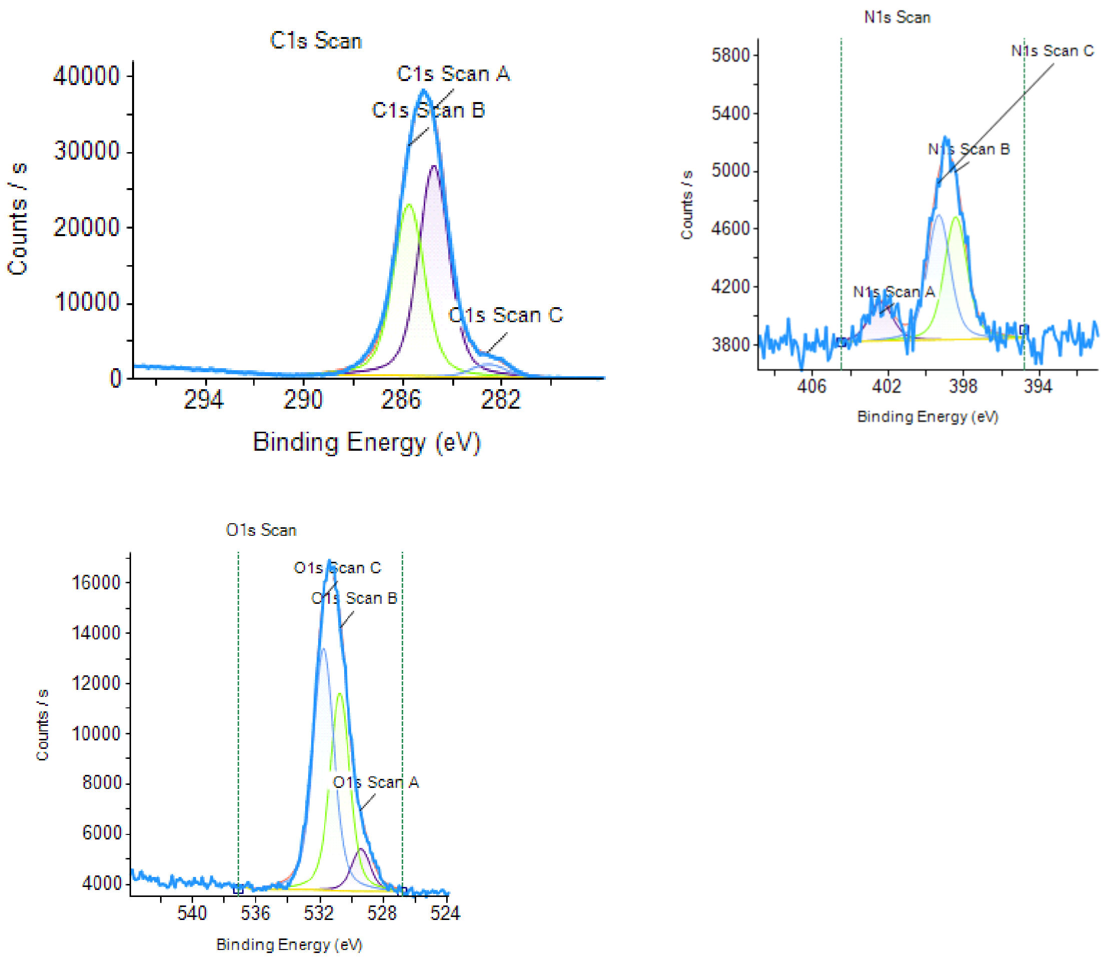

Figure S5. C1s, N1s, and O1s XPS spectra of initial DND sample for Mn-DND.

Table S5. Elemental ID and Quantification in initial and Mn-DND samples.

Sample	***Level***	***Peak BE***	***Atomic %***
Initial	C1s	284.8	86.2
	N1s	398.5	2.0
	O1s	530.9	11.8
Mn-DND	C1s	284.7	85.4
	N1s	398.4	2.5
	O1s	530.9	12.0
	Mn2p	640.4	0.1%

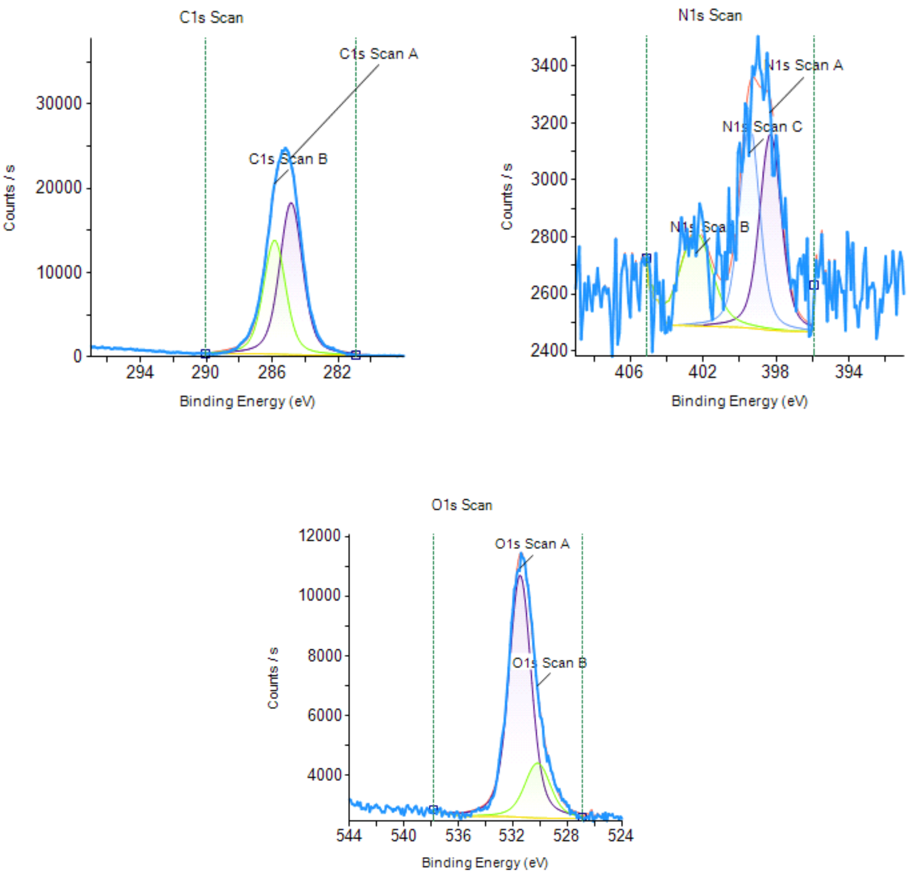

Figure S6. C1s, N1s, and O1s XPS spectra of Mn-DND.

Table S6. Detail Quantification of C1s, O1s, and N1s peaks in initial DND and Mn-DND

Compound	***C1s peak***	***C1s, at%***	***Group***	***Reference***
Initial DND	282.6	3.8	Impurity carbide Fe_3C	[1-6, 12]
	284.8	52.6	C-C sp^3	
	285.8	43.8	C-C, C-N, C-H	
Mn-DND	284.8	57.8	C-C sp^3	
	285.9	42.2	C-C, C-N, C-H	
Compound	***O1s peak***	***O1s, at%***	***Group***	[1-6]
Initial DND	529.4	8.2	COOH	
	530.7	40.2	C-O	
	531.8	51.6	C=O carbonyl	
Mn-DND	529.4	8.2	COOH	
	530.2	20.8	C-O	
	531.5	79.0	C=O carbonyl	
Compound	***N1s peak***	***N1s, at%***	***Group***	[7-11]
Initial DND	398.4	42.5	N-sp^3C	
	399.3	43.4	N-H, C=N-C	
	402.4	14.1	N-O	
Mn-DND	398.3	37.2	N-sp^3C	
	399.5	36.4	N-H, C=N-C	
	402.4	26.4	N-O	

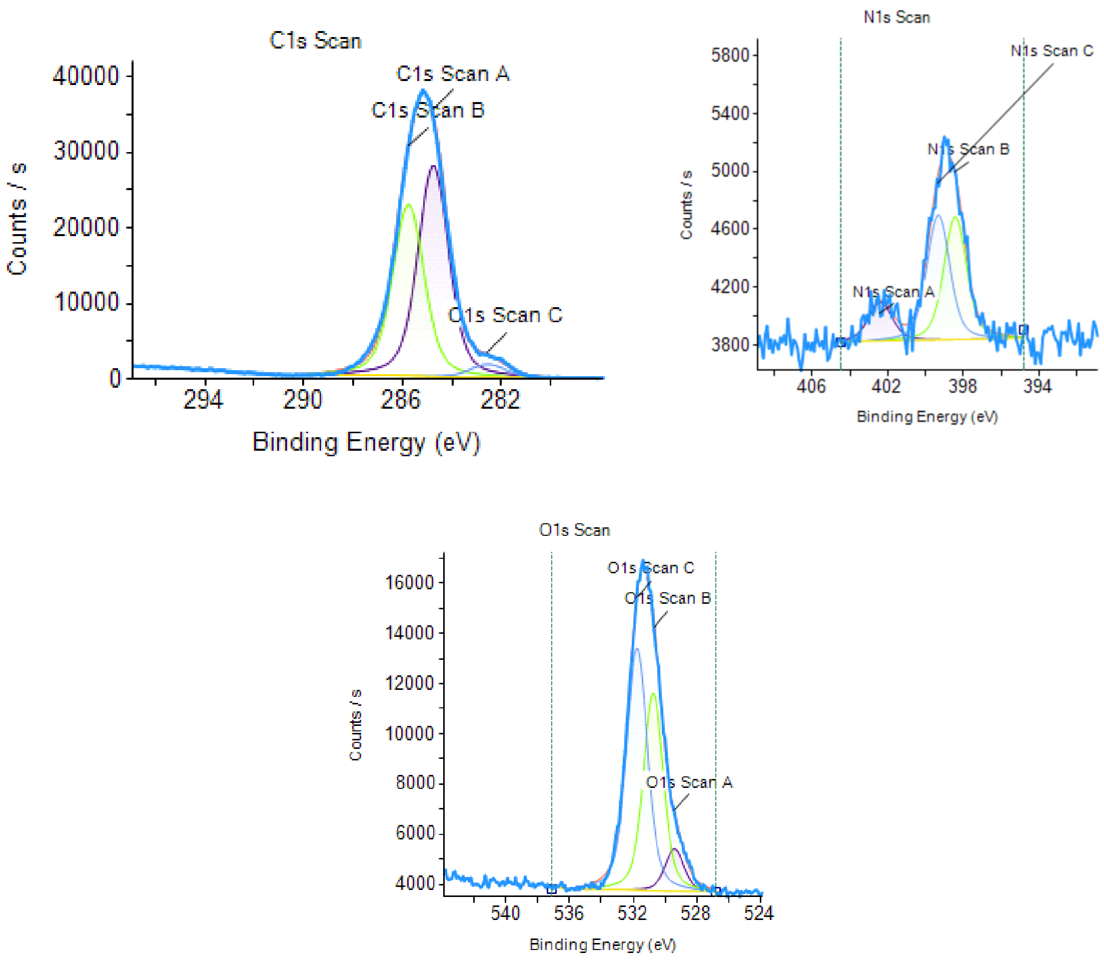

Figure S7. C1s, N1s, and O1s XPS spectra of initial DND sample for Gd-DND.

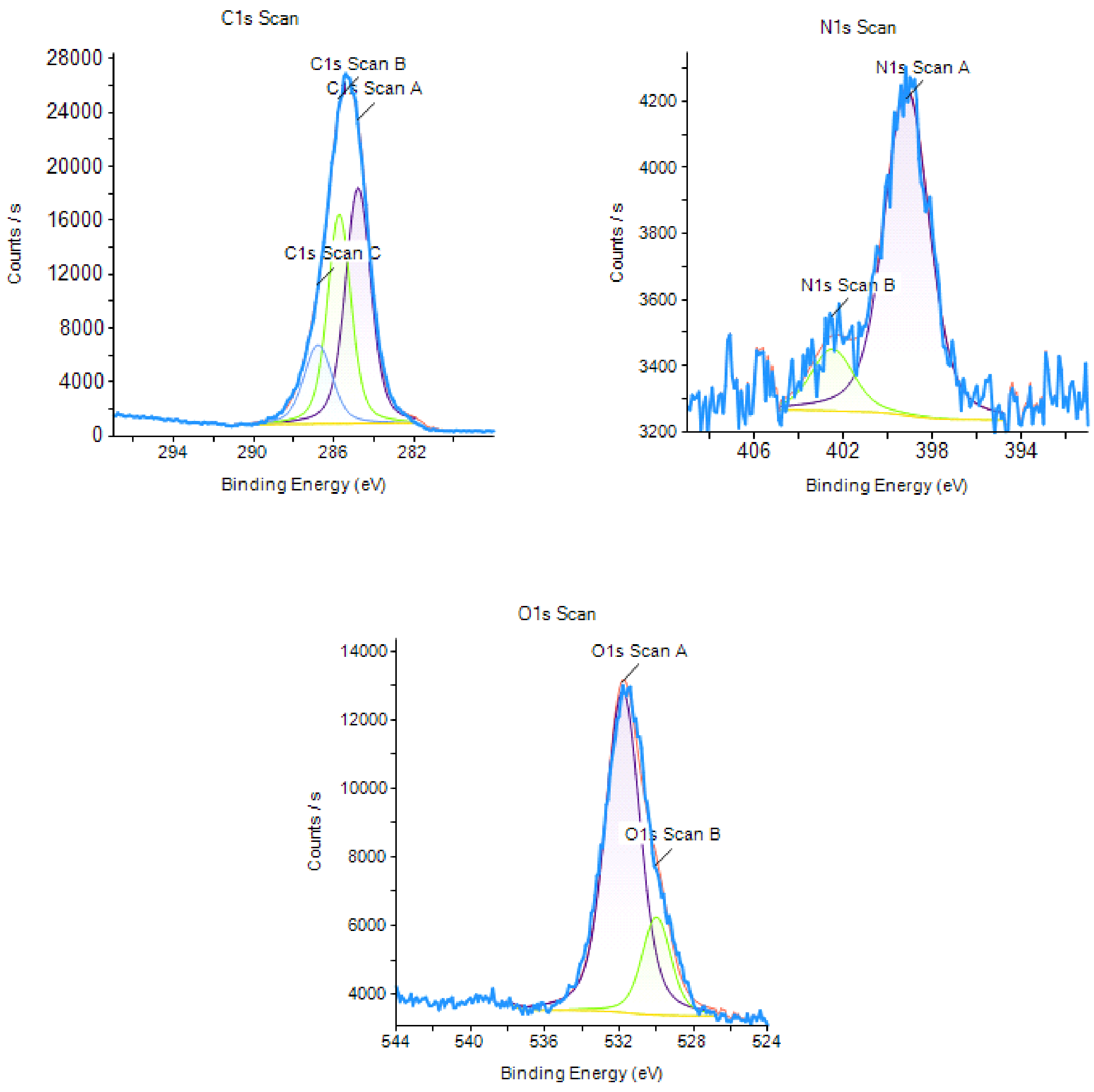

Figure S8. C1s, N1s, and O1s XPS spectra of Gd-DND.

Table S7. Elemental ID and Quantification in Gd-DND

Sample	***Level***	***Peak BE***	***Atomic %***
**Initial**	C1s	284.8	86.2
	N1s	398.5	1.9
	O1s	530.9	11.9
**Gd-DND 4d**	C1s	284.8	83.3
	Gd4d	140.5	0.1
	N1s	398.2	2.0
	O1s	530.9	14.6
**Gd-DND 3d**	C1s	284.8	83.5
	Gd3d5	1185.8	0.2
	N1s	398.7	2.3
	O1s	531.1	14.0

Table S8. Detail Quantification of C1s, O1s, and N1s peaks in initial DND and Gd-DND

Compound	***C1s peak***	***C1s, at%***	***Group***	***Reference***
Initial DND	282.6	3.7	Impurity carbide Fe_3C	[1-6, 12]
	284.8	52.6	C-C sp^3	
	2858	43.7	C-C, C-N, C-H	
Gd-DND, Gd3d,4d	284.8	45.5	C-C sp^3	
	285.7	37.4	C-O, C-N	
	286.8	17.1	N-C=O, O=C-O	
Compound	***O1s peak***	***O1s, at%***	***Group***	[1-6]
Initial DND for Gd	529.4	8.2	COOH	
	530.7	40.2	C-O	
	531.8	51.6	C=O carbonyl	
Gd-DND Gd3d, 4d	530.0	20.3	COOH	
	531.8	79.7	C=O carbonyl	
Compound	***N1s peak***	***N1s, at%***	***Group***	[7-11]
Initial DND for Gd	398.4	42.5	N-sp^3C	
	399.3	43.4	N-H, C=N-C	
	402.4	14.1	N-O	
Gd-DND Gd3d, 4d	399.1	84.7	N-H, C=N-C	
	402.5	15.3	N-O	

Typical experimental error in the XPS determination of atomic ratios of paramagnetic ions is around 20%.

References

1. Nunn, N.; d'Amora, M.; Prabhakar, N.; Panich, A.M.; Froumin, N.; Torelli, M.D.; Vlasov, I.; Reineck, P.; Gibson, B.; Rosenholm, J.M.; Giordani S.; Shenderova, O. Fluorescent single-digit detonation nanodiamond for biomedical applications. *Methods Appl. Fluoresc*. **2018**, *6*, 35010. https://doi.org/10.1088/2050-6120/aac0c8

2. Shenderova, O.; Panich, A.M.; Moseenkov, S.; Hens, S.C.; Kuznetsov, V.; Vieth, H.M. Hydroxylated Detonation Nanodiamond: FTIR, XPS, and NMR Studies. *J. Phys. Chem. C* **2011**, *115*, 19005–19011. dx.doi.org/10.1021/jp205389m

3. Albers, P.W.; Klein, H.; Lox, E.S.; Seibold, K.; Prescher G.; Parker, S.F. INS-, SIMS- and XPS-investigations of diesel engine exhaust particles. *Phys. Chem. Chem. Phys*. **2000**, *2*, 1051-1058. DOI https://doi.org/10.1039/A909070C

4. Gaur, P.; Banerjee, S. C-N cross coupling: Novel approach towards effective aryl secondary amines modification on nanodiamond surface. *Diamond Relat. Mater*. **2019**, *98*, 107468. https://doi.org/10.1016/j.diamond.2019.107468

5. Haasz, A.A.; Chiu, S.; Pierre, J.E.; Gudimenko, Y.I. Thermo-oxidative erosion of amorphous hydrogenated carbon films. *J. Vac. Sci. Technol. A* **1996**, *14*, 184-193. https://doi.org/10.1116/1.579916

6. Sharin, P.P.; Sivtseva, A.V.; Popov, V.I. Composition and Chemical State of Nanopowder Particles Obtained by Grinding Natural Diamond and by Detonation Synthesis. 2021 *IOP Conf. Ser.: Mater. Sci. Eng.* **2021**, *1079*, 52023.

7. Greczynski, G.; Hultman, L. C1s Peak of Adventitious Carbon Aligns to the Vacuum Level: Dire Consequences for Material's Bonding Assignment by Photoelectron Spectroscopy. *ChemPhysChem* **2017**, *18*, 1507 – 1512. https://doi.org/10.1002/cphc.201700126

8. Crunteanu, A.; Charbonnier, M.; Romand, M.; Vasiliu, F.; Pantelica, D.; Negoita, F.; Alexandrescu, R. Synthesis and characterization of carbon nitride thin films obtained by laser induced chemical vapour deposition. *Surface Coatings Technol*. **2000**, *125*, 301–307. https://doi.org/10.1016/S0257-8972(99)00577-0

9. Egelhoff, Jr., W.F. N_2 on Ni(100): Angular Dependence of the N_{1s} XPS Peaks. *Surf. Sci. Lett*. **1984**, *141*, L324-L328. https://doi.org/10.1016/0039-6028(84)90131-6

10. Marton, D.; Boyd, K.J.; Al-Bayati, A.H.; Todorov, S.S.; Rabalais, J.W. Carbon nitride deposited using energetic species: a two-phase system. *Phys. Rev. Lett*. **1994**, *73*, 118–121. DOI: https://doi.org/10.1103/PhysRevLett.73.118

11. Souto, S.; Alvarez, F. The role of hydrogen in nitrogen-containing diamond-like films studies by photoelectron spectroscopy. *Appl. Phys. Lett*. **1997**, *70*, 1539–1541. https://doi.org/10.1063/1.118611

12. Aleksenskii, A.E.; Osipov, V.Yu.; Vul', A.Ya.; Ber, B.Ya.; Smirnov, A.B.; Melekhin, V.G.; Adriaenssens, G.J.; Iakoubovskii, K. Optical properties of nanodiamond layers. *Phys. Solid State* **2001**, *43*, 145–150.
